# Students' perceptions on how e-learning platforms in universities should be improved to increase the quality of educational services

**DOI:** 10.25122/jml-2023-0274

**Published:** 2023-09

**Authors:** Dodu Gheorghe Petrescu, Cristina Neculau, Aida Geamanu, Mihai Adrian Dobra, Ana-Maria Nedelcu, Alin Gabriel Sterian

**Affiliations:** 1 Department of Marketing and Medical Technology, University of Medicine and Pharmacy Carol Davila Bucharest, Bucharest, Romania; 2 Department of Ophthalmology, University of Medicine and Pharmacy Carol Davila Bucharest, Bucharest, Romania; 3 Center of Uronephrology and Renal Transplant Fundeni, University of Medicine and Pharmacy Carol Davila Bucharest, Bucharest, Romania; 4 Romanian Academy, Institute of National Economy, Bucharest, Romania; 5 Department of Pediatric Surgery and Orthopedics, University of Medicine and Pharmacy Carol Davila Bucharest, Bucharest, Romania; 6 Emergency Hospital for Children Grigore Alexandrescu, Bucharest, Romania

**Keywords:** eLearning, Covid-19, medical education, education 5.0, remote education, online education

## Abstract

The Covid-19 pandemic has made a strong mark on the education system globally. Universities have been compelled to take urgent measures to provide young people with access to education, leading them to invest significant resources in building their own e-learning platforms. Because they were created and implemented in a relatively short time, certain e-learning platforms presented several issues, underscoring the need for improvement. This paper aims to identify students' perceptions of how e-learning platforms should be refined in order to increase the quality of online educational services. The research involved a sample of 114 students enrolled in the University of Medicine and Pharmacy Carol Davila in Bucharest. Data collection was conducted using an online questionnaire, and data analysis was performed using the IBM SPSS software. The results indicated that there is room for improvement in the quality of the services offered. To achieve this, further steps should focus on the interface of the online platforms, optimizing the presentation of educational materials, and refining the communication between teachers and students. Furthermore, other online tools (e.g., blogs, forums, or mobile applications) should be incorporated within these platforms, in order to facilitate the integrative distribution of information.

## INTRODUCTION

The COVID-19 pandemic has played a very important role in the development of online learning worldwide. While in the past educational establishments relied mostly on traditional education (where the teaching-learning process took place face-to-face), the passage of time has led to the adoption of online education, enabling data sharing, faster communication between students and teachers, and faster access to resources. During the COVID-19 pandemic, the urgent need to create and implement such e-learning platforms gradually generated certain issues, which made some students dissatisfied with the teaching-learning process.

E-learning evolved in tandem with the technological advancements at the beginning of the 21^st^ century, becoming a multidimensional concept that includes the economic, educational, and institutional levels [1]. E-learning is perceived as an innovative teaching and learning method that is constantly improving, based on Information Technology and Communications (IT&C) to provide access to information resources, manage them without time and space constraints, and promote student interaction through dedicated digital tools such as educational platforms [2]. In recent years, e-learning has also been increasingly used in medical educational institutions to facilitate the transfer and assimilation of information [3]. The use of new technologies in this field is of great importance as it illustrates the efforts at the national and international levels to achieve sustainable development in both health and education [4].

Students need digital skills to find, evaluate, and use information in the digital environment [5]. Online platforms aim to create an interactive learning environment centered on the student, ensuring an individualized learning process and permanent access to information resources [6]. The technological structure is an essential condition for the proper functioning of e-learning platforms. Connectivism is a theory of the educational process that has developed in the digital age and shows the role of the socio-cultural context in which learning occurs. The educational process takes place through online networks. Technology is the main driving force behind e-learning and guarantees the overcoming of existing educational boundaries, to ultimately create an unlimited horizon of educational development [7].

E-learning has many advantages compared to traditional education, such as lower costs, elimination of time and space limitations, flexibility, student-centered tailored education, or improvement of the learning process. Education 5.0 reflects the fifth industrial revolution in education [8]. According to specialists in this field, Education 5.0 aims at the professional development of individuals, using original thinking to identify solutions to existing problems as well as promote a learning culture based on a set of values such as emotional intelligence and social awareness [9]. Through the use of technological tools, Education 5.0 provides tailored educational experiences and keeps students interested. Artificial intelligence algorithms are used to assess students' learning styles and customize their learning materials, while virtual and augmented reality provide interactive experiences and understanding of a given environment.

Education 5.0 [10, 11] emphasizes real-world experiences and facilitates learning opportunities in any setting and at any time. Through the use of ever-evolving technological tools, students can understand the subjects of study from their own experiences. Focusing on individuals rather than technologies, [12], Education 5.0 [13] aims to harness the potential of individual learning by monitoring learning styles and providing adjusted educational materials. Furthermore, it strives to foster learning through one's own experiences in real-world settings, using various digital tools. Education 5.0 [14] is the future of education and promises to deliver a dynamic and intuitive experience that piques the interest of both the student and their teacher. With the right tools and environment, Education 5.0 has the potential to revolutionize the way we learn.

The Covid-19 pandemic had a strong impact on educational activity [15]. Universities had to implement new measures to facilitate students' access to teaching materials [16] and to streamline the process of assimilating information. Thus, during the pandemic they had to create their own online platforms [17], improve their online tools, and try to create integrated platforms to enable quick and easy access to information [18, 19]. Although educational establishments have made a substantial effort to provide students with all the necessary learning materials, time has illustrated that they have also generated some problems in terms of their use. Problems were encountered with the log-in process, with waiting times to access and download certain materials, with the communication process between teachers and students, and with the possibility of sharing documents with remote persons.

All these issues have led those managing online activity in universities to analyze the existing situation and take the necessary steps to address the weaknesses of these online tools.

Based on these aspects, we considered it necessary to conduct a quantitative study to identify the perception of students enrolled in the University of Medicine and Pharmacy Carol Davila in Bucharest on the measures that should be taken within the university to improve the quality of online education services.

## MATERIAL AND METHODS

Based on the weaknesses of e-learning platforms that were identified during the Covid-19 pandemic, a survey identifying the main measures that should be taken to enhance the learning activity on e-learning platforms was carried out between April and May 2022 at the University of Medicine and Pharmacy Carol Davila in Bucharest. The research was conducted on 114 students using a questionnaire that was posted on an online platform and subsequently distributed to respondents.

## RESULTS

The first aspect studied in this research concerns the identification of students' perceptions of the main online tools that they would improve within the university in order to increase the quality of educational services. For this purpose, a profile of the respondents would be relevant, as shown in Table 1, which contains useful data for further research such as the respondents’ preferred form of education, age, gender, and residence.

**Table 1:** Profile of the respondents

	Category	Frequency	Percentage (%)
**Respondents’ preferred form of education**	Text-based course	57	50
	Animated examples	12	10.5
	Simulations	17	14.9
	Project activities	5	4.4
	Graphical examples	10	8.8
	Video clips	9	7.9
	Case study solving	4	3.5
**Age**	18-24 years old	100	87.7
	25-35 years old	6	5.3
	36-45 years old	8	7
**Gender**	Men	15	13.2
	Women	99	86.8
**Residence**	City	98	86
	Countryside	16	14

Regarding the profile of the respondents who participated in the research, it should be noted that out of the total of 114 students, 50% of them indicated that they prefer text-based courses. Moreover, 14.9% mentioned that their preferred learning method is simulation-based, while 10.5% of them prefer animated examples, considering that they are able to better illustrate how certain medical procedures are performed. A smaller proportion of those who participated in this research (8.8%) prefer to assimilate information from graphic examples provided by teachers, while 7.9% prefer videos. Additionally, 4.4% of the students mentioned that they learn better from project-based activities while 3.5% of them prefer case studies.

Analyzing the profile of the respondents in terms of demographic variables, 86.8% of the students are female while 13.2% are male. Regarding the age distribution, 87.7% are aged between 18-24 years, 7% between 36-45 years, and 5.3% of them are aged between 25- 35 years. Studying the profile of the respondents in terms of distribution according to the respondents' residence environment, it can be observed that 86% are from urban areas, while, 14% of them are from rural areas.

Table 2 presents students’ perceptions regarding online tools provided by the university in order that they would improve in order to reach a more effective online learning environment. As shown below, the analyzed online tools that students would enhance are the university’s website, digital library, e-learning platform, the students’ forum, and mobile applications used within the university as well as social media.

**Table 2:** Students' perception of online tools within the university that they would improve to increase the quality of online learning

Online tools	Frequency	Percentage (%)
University website	16	14
The digital library of the university	41	36
The E-learning platform of the university	12	10.5
Students Forum	6	5.3
Mobile applications used within the university	15	13.2
Social media activity	24	21.1

Following data analysis, 36% of the respondents indicated that they would improve the digital library currently existing in the educational institution, while 21.1% of them indicated that more importance should be given to social networking activities. It can thus be seen that they would like on the one hand to have access to educational resources of a much higher quality, but on the other hand, they would also like to improve the process of communication with teachers, other students, or specialists outside the university.

Furthermore, 13.2% of students would like to improve the mobile applications that are used within the university, 14% of them believe that the university website should be revised, and 10.5% of respondents believe that more emphasis should be put on improving the e-learning platform. A smaller proportion of respondents (5.3%) indicated that a number of actions should be implemented to help optimize the student forum.

Table 3 presents the areas of improvement for the e-learning platform of the university. The interface of the platform, the way in which dedicated teaching materials are posted on the platform, the communication between teachers and students as well and the way in which students communicate with each other represent important elements that should be taken into consideration for our study.

**Table 3:** Areas for improvement of the university's e-learning platform

Areas that could be improved	Frequency	Percentage (%)
E-learning platform interface	33	29.8
How teaching materials are posted to the online platform	29	25.4
Communication between teachers and students	29	25.4
The way students communicate with each other	23	20.2

Following data analysis, 29.8% of the respondents indicated that the interface of the e-learning platform is the first aspect that needs to be addressed. Most likely, they consider that it should be easier and more intuitive, to facilitate access to information.

Furthermore, 25.4% of respondents indicated that both the way materials are posted on the e-learning platform and the communication process between teachers and students should be improved. The students believe that the teaching process should be upgraded, with a strong emphasis on the way information is distributed from teachers to students and the way educational materials are shared on the platform. Additionally, 20.02% of students think that the communication process between students should be adjusted in order to make the learning process more effective.

Table 4 presents the elements that students would add to the e-learning platform of the university in order to increase its effectiveness. These are represented by online tools such as adding a blog containing case studies made by medical specialists, implementing a debate forum on relevant topics for the students, the addition of 2D and 3D animations in order to create a better learning environment, and access to the virtual library to be made directly from the online platform.

Regarding the elements that respondents would add to the university's e-learning platform to increase its efficiency, the analysis showed that 32.5% of participants indicated that they would add a blog where case studies and research by other specialists in the field would be constantly posted.

**Table 4:** Elements that students would add to the university's e-learning platform to increase its effectiveness

Online tools	Frequency	Percentage (%)
Adding a blog to the e-learning platform to post case studies by medical specialists	37	32.5
Implementation of a forum to debate several topical issues	35	30.7
Adding 2D and 3D animations to facilitate the assimilation of certain information	31	27.2
Access to the virtual library directly from the platform	11	9.6

Further on, 30.7% of respondents indicated that they would implement a forum where there would be an opportunity to debate a range of topical issues. In addition, students would have the possibility to find out current information in various fields of activity or to identify solutions to certain problems. To improve the educational process on the e-learning platform, 27.2% of the respondents indicated that they would add a series of 2D and 3D animations explaining certain aspects that they study in practice. A smaller proportion of respondents (9.6%) would add to the platform the possibility to directly access the university's digital library, in order to have all the online tools used in the educational establishment integrated.

Table 5 presents the measures that are proposed by students in order to increase the quality of online learning within the university. These measures concern conducting online courses with foreign doctors, as well as specialists from both public and private medical institutions, holding debates with external participants, and holding online events in which students can discuss topics of interest.

Regarding the main measures that should be taken within the university to increase the quality of online education, 31.6% of the respondents mentioned that courses should be conducted on the e-learning platform with foreign medical professionals to identify their opinions on certain topical issues. In addition, they considered it necessary to hold debates on this platform so that young students could be more involved in discussions and learn from each other.

**Table 5:** Measures proposed by respondents to increase the quality of online learning at the university

Proposed measures	Frequency	Percentage (%)
Conducting online courses with foreign doctors	36	31.6
Conducting online courses with various specialists working in both public and private medical units	24	21.1
Holding debates on the e-learning platform with the participation of people from outside the university	36	31.6
Holding online events for students to discuss topics	18	15.8

In order to improve the quality of online education in the university 21.1% of the respondents considered that a series of online courses should be implemented with specialists working in both public and private medical units, while 15.8% shared the opinion that a series of online events would help students (conferences, seminars, etc.) better assimilate information topics.

Table 6 illustrates the m-health applications that should be used in conjunction with other online tools in order to improve the quality of online learning within the university. The m-health applications that we considered to be the most relevant for our study are Medscape, Brainscape, Prognosis: Your Diagnosis, and Merck Sharp & Dohme (MSD) Manual Profesional. We considered other applications as well if some of the students did not utilize the ones mentioned above or considered others to be more effective.

**Table 6:** M-Health applications that should be used in the university in conjunction with other online tools to increase the quality of online learning

M-health applications	Frequency	Percentage (%)
Medscape	39	34.2
Brainscape	25	21.9
Prognosis: Your Diagnosis	19	16.7
MSD Manual Professional	13	11.4
Other applications	18	15.8

Regarding the mobile apps that should be implemented at the university level to be used in conjunction with the other online tools, 34.2% of students preferred the Medscape app, while 21.9% of them mentioned the Brainscape app. Additionally, 16.7% of the respondents opted for the Prognosis: Your Diagnosis app while 11.4% of them indicated that the MSD Manual Professional app should be used. Other mobile applications that should be used within the university to increase the quality of online education services were mentioned by 15.8% of the respondents.

## DISCUSSION

E-learning involves the use of digital technologies to facilitate the transfer of knowledge at the pedagogical level [20]. The new technologies used in education play a very important role in the teaching-learning process, as they provide students access to a large number of resources and enable them to obtain personalized educational materials, thus contributing to a high extent to their professional training [21].

Online learning was used extensively during the COVID-19 pandemic when a number of online tools were developed that allowed teachers to connect with remote students. Although the initial transition from traditional to online learning was difficult, specialists implemented several upgrades to the latter, which enhanced the way education is conducted in the digital environment.

This study aimed to identify how e-learning platforms can be adjusted in order to increase the quality of educational services offered in the university. The analysis showed that, in terms of web tools that should be improved at the university level, most students considered that the digital library (36%), social networking activity (21.1%), and the university website (14%) should be reviewed. In order to increase the quality of online services offered in the university, courses with doctors from outside the country should be carried out as well as debates on various topics, so that students have the opportunity to learn from each other (31.6%). Moreover, respondents were of the opinion that courses with specialists working in both public and private environments (21.1%) could help students to assimilate information more easily. Regarding the university's e-learning platforms, students felt that the interface of the platform should be improved (28.9%), as well as the way teaching materials are posted, and the communication process between teachers and students (25.4%).

They reported that in order to enhance the activity on the online platform, a blog (32.5%) and a forum (30.7%) should be developed to support open debates. Respondents also considered it necessary to implement mobile applications within the university to help young people better assimilate the information taught. These include Medscape (34.2%), Brainscape (21.9%), and Prognosis: Your Diagnosis (16.7%).

Regarding the limitations of this research, it should be noted that the first aspect refers to the limited number of respondents (N=114). In the future, other studies should evaluate a larger number of students to allow extrapolation of the results to the entire community. Another limitation concerns the narrow number of variables taken into account. Another limitation concerns the way in which the questionnaire was distributed to respondents. It was posted on an online platform and subsequently made available to respondents. In the future, in order to register a higher number of respondents it should be distributed both in physical and online format.

## CONCLUSION

E-learning platforms play a very important role in educational establishments. They allow teachers to post teaching materials needed for the learning process, to discuss and keep constant communication with students, and to give lectures/seminars in digital format. In addition, they can have various guests (specialists from the country or abroad) who can share with the students some of their expertise in various fields of interest. While in the COVID-19 pandemic, online tools were used extensively to run the teaching process in optimal conditions, they are now used to support the traditional process. Even now, teachers are posting on e-learning platforms the information young people need.

Considering the results of this quantitative study, it is believed that future qualitative studies (e.g., focus groups, and in-depth interviews) should further explore this topic. In addition, such research would provide a more comprehensive picture of the subject and help practitioners identify other aspects that could be improved in e-learning platforms. Moreover, in order to better understand and have a better overview of this topic, further quantitative studies should be carried out at several universities in the country.

## Personal thanks

Publication of this paper was supported by the University of Medicine and Pharmacy Carol Davila, through the institutional program Publish not Perish.
